# The Effects of Having a Child with Cancer on Parental Psychology: A Cross-Sectional Study

**DOI:** 10.3390/jcm13196015

**Published:** 2024-10-09

**Authors:** Begum Sirin Koc, Funda Tekkesin, Aysenur Kanat Aydin, Zehragul Balik, Ulku Miray Yildirim, Selime Aydogdu, Suar Caki Kilic

**Affiliations:** 1 Department of Pediatric Hematology and Oncology, Umraniye Research and Training Hospital, University of Health Sciences, 34764 Istanbul, Turkey; tekkesinfunda@gmail.com (F.T.); ulku-miray@hotmail.com (U.M.Y.); selimea69@hotmail.com (S.A.); suarck75@gmail.com (S.C.K.); 2 Department of Psychology, Umraniye Research and Training Hospital, University of Health Sciences, 34764 Istanbul, Turkey; kanataysenur@gmail.com (A.K.A.); zehrabalik@gmail.com (Z.B.)

**Keywords:** cancer, child, parent, pediatric oncology, psychology

## Abstract

**Objective**: In childhood cancers, parents are affected psychologically as well as children. We aimed to evaluate the effect of childhood cancer type and stage, as well as elapsed time from diagnosis, on the anxiety and stress indicators among parents. **Materials and Methods**: Patients aged between 0–18 years and diagnosed with cancer who were receiving chemotherapy (0–1 month, 1–6 month, 6–12 month) and completed treatment were included. Parents of those children (mother or father) who agreed to participate this study were also included. The personal information form and three psychological assessment scales (Beck Hopelessness Scale (PSS), Psychological Resilience Scale (PRS), Uncertainty Intolerance Scale (IUS)) were used for assessment of mental health of the parents. Scores of the scales and clinical features of the children with cancer were compared statistically. **Results**: The study included 84 parents (57 mothers, 27 fathers) and 84 children. The rate of children with solid tumors was 60% (*n*:50) and 40% of them were metastatic, which means advanced stage. The rate of the children with leukemia was 40% (*n*:34) and 23% of them were in high-risk group. According to the type (leukemia vs. solid tumors) and stage (high risk/advanced stage vs. others) of the cancer, there was no statistical difference among parents in the psychological scales (*p* > 0.05). Additionally, results of the psychological scales showed no significant difference between mothers and fathers (*p* > 0.05). The hopelessness scores are significantly higher among parents whose child’s treatment is terminated compared with those whose active therapy is ongoing, and resilience scores are higher among parents who have less than 1 month since diagnosis of childhood cancer than later periods. **Conclusions**: The regular assessment of mental health among parents of children with cancer across all the survivorship trajectory: at the time of diagnosis, during active therapy, and after treatment.

## 1. Introduction

Approximately 400,000 children are diagnosed with cancer every year [1]. Expected 5-year life expectancy among these cancer types varies from over 80% to below 20%, depending on the cancer type and stage [2]. In Turkey, the most common childhood cancers are acute leukemias, lymphomas, brain tumors, and other solid tumors [3]. In the country, cancer is the fourth leading cause of childhood death after infections, accidents, and cardiac deaths [4]. Through the advances in cancer diagnosis and treatment over the years, today the 5-year relative survival rate of cancer has increased from 58% to 84% for children and from 68% to 85% for adolescents [5].

However, parents experience a difficult process both physically and psychologically during the treatment process [6]. Following diagnosis, parents quickly learn a significant amount of information about cancer, adopt specific caregiving skills, and realign family roles and routines. However, this transition to the new role is not smooth and can lead to experiencing significant emotional strain that can persist over time for many parents [7,8].

Parents are affected physically, psychologically, socially, spiritually, and economically due to the life-threatening nature of cancer and the long and difficult treatment process [9,10,11]. In the literature, being diagnosed with cancer is a crucial stress factor in adult patients, and it is considered a ‘traumatic event’ [12]. Parents of children with cancer also describe it as a ‘life-changing experience’ [13]. Studies emphasize the importance of early identification of psychological risk factors for family members who require psychological help in the process. Psychological support interventions for parents and caregivers can be beneficial, especially in families with young children and infants [14]. The aim of this study was to compare the stress and anxiety levels among parents of children diagnosed with cancer and to evaluate the effect of type and stage of cancer and time elapsed since diagnosis on stress and anxiety in parents.

## 2. Methods

This cross-sectional study was conducted in a pediatric hematology and oncology clinic with a medical team including pediatric oncologists, nurses, two psychologists, a spiritual support specialist, and a play therapist. This team treats almost 100 children with newly diagnosed cancer each year.

### 2.1. Patients

Patients aged between 1–18 years and who were diagnosed with cancer such as leukemia, lymphoma, brain tumors, or other solid tumors were included. They were grouped according to the elapsed time of diagnosis, and whether they were receiving chemotherapy at 0–1 month, 1–6 months, and 6–12 months after diagnosis or had completed a treatment protocol, which was followed-up at the outpatient clinic. The patients who stayed in hospital and were receiving chemotherapy in the first month were accepted as newly diagnosed.

### 2.2. Parents

Parents of children who were diagnosed with malignancy such as leukemia, lymphoma, brain tumors, or other solid tumors and receiving chemotherapy (in the first month, 1–6 months, 6–12 months) or completed treatment were included. We excluded the parents who had children who had relapsed or died due to cancer, since their psychology may have been strongly different from other parents. Parents not willing to participate to this study were excluded. One parent (mother or father) of each child was enrolled. Parents first filled out the information consent form. The parents were interviewed during the treatment or after completion of therapy of their children.

### 2.3. Study Design

Over a period of six months, between April 2020–January 2021, we collected data on the sociodemographic status (age of parents, number of siblings, whether they receive support from family members, financial status, education level, employment status, and marital status) of the parents using the personal information form. The clinical information of the patients (the age, the gender, the elapsed time of diagnosis, the type of cancer, the stage of cancer or the risk group for children with leukemia, and whether receiving chemotherapy) were recorded.

We assessed the mental health of these parents using three psychometric scales to measure depression and anxiety: the Beck Hopelessness Scale (BHS) (20 questions), Intolerance to Uncertainty Scale (IUS) (26 questions), and Psychological Resilience Scale (PRS) (33 questions). We used three scales consisting of a total of 79 questions. The reliability and validity studies of these scales were previously performed in the country [15,16,17].

- The Beck Hopelessness Scale (BHS)

The BHS is designed to assess the subjective aspects of depression: it is a subjective measure of depression, consisting of 20 items. For each item, the score ranges from zero to one. The higher the global score, the more depressed the subject is (0 to 20). It alerts the clinician to diagnosis of depression from a score of four and helps to judge the intensity of this depression:

- 0 to 3: minimal
- 4 to 8: mild
- 9 to 14: moderate (close follow-up)
- 15 and over: severe (definite suicidal tendency)

- Intolerance to Uncertainty Scale (IUS)

The IUS evaluates emotional, cognitive, and behavioral reactions to ambiguous situations, implications of being uncertain, and attempts to control the future. It is one of the rating scales developed to measure the severity of anxiety symptoms. The scale consists of 26 items, each defined by a series of symptoms, and measures anxiety. We added up the responses for each of the items to score the IUS as a unifactorial tool.

- Psychological Resilience Scale (PRS)

The PRS consists of 33 items and is based on 5 intrapersonal and interpersonal dimensions that measure personal protective factors that promote adaptation to challenges.

### 2.4. Statistical Analysis

All data were recorded on the SPSS program and compared statistically. The t-test for independent groups and the Mann–Whitney U test were used to compare the scores of the Beck Hopelessness Scale, the Intolerance of Uncertainty Scale, and the Psychologically Resilience Scale. The Kruskal–Wallis H Test was used to compare the parents’ BHS and PRS scores according to the time elapsed after their children were diagnosed (0–1 month/1–6 months/6–12 months/1 year). One-way analysis of variance (ANOVA) was performed to compare parents’ BHS scores according to the time elapsed after diagnosis (0–1 month/1–6 months/6–12 months/1 year). In the analysis of psychological scales with the risk of status of cancer, Kruskal–Wallis H Test and Post Hoc (Tamhane Test) were also used.

According to the risk status of cancer, there was no significant difference in the IUS, BHS, and PRS scores of the parents.

Spearman correlations were used in the relationship between socio-demographic characteristics and scores obtained from psychological scales. *p* ≤ 0.05 was considered statistically significant.

### 2.5. Ethical Statement

The study was reviewed and approved by the Ethics Committee of our hospital (approval date and number: 19 March 2020 and B.10.1.TKH.4.34.H.GP.0.01/67). A consent form was obtained from the parents by the researchers.

## 3. Results

### 3.1. Parents Characteristics

There was a total of 320 eligible parents of children with cancer. Overall, 84 parents (57 mothers and 27 fathers) voluntarily participated in the study. We excluded the parents who had children were relapsed (*n*:27) or died (*n*:16) due to cancer. The remaining parents (*n*:236) declined to participate into the study. Most of the parents were mothers (68%) and the majority of parents were aged between 30 and 49 years. All the parents were Turkish. The socio-demographic characteristics of the parents (age, gender, number of children in the family, job status, and household income level) were summarized in Table 1.

### 3.2. Patient Characteristics

Most of the children (*n*:66, 78%) were under 13 years old, and half of them (*n*:42) were 0–6 years old. All the children (*n*:84) were treated with chemotherapy with/without surgery. At the time of the study, more than half of the children (*n*:54, 64%) were actively receiving treatment and the others (36%, *n*:30) had finished their treatment. According to the time elapsed after cancer diagnosis, 23 patients were within 1 month, 26 patients within 1–6 months, 13 patients within 6–12 months, and 22 patients within >1 year. Over half of the children (*n*:50, 60%) had solid tumors and 40% of them were metastatic (advanced stage) at diagnosis. Approximately half of children (*n*:34, 40%) were diagnosed with leukemia; the risk groups of children with leukemia were determined according to the ALLIC-BFM 2009 Protocol. The majority of children with leukemia were in the moderate-risk (*n*:22, 64.7%) and high-risk (*n*:8, 23.5%) groups. The distribution of diagnoses, risk groups, and age groups of the children are presented in Table 2.

### 3.3. Analysis of the Psychological Scales of the Parents

In the correlation analysis between the scales, it was found that there was a negative correlation between the scores of the Beck Hopelessness Scale and the scores of the Psychological Resilience Scale, and a positive correlation with the scores of the Intolerance to Uncertainty Scale (Table 3). No significant difference was observed between mothers and fathers regarding the variables of hopelessness, resilience, and intolerance to uncertainty scales (*p* > 0.05).

The scores of the hopelessness, resilience, and uncertainty intolerance scales were compared between the parents of children with leukemia and the parents of children with other cancers; no statistically significant difference was found (*p* > 0.05). However, it was determined that the parents of children with leukemia had significantly lower scores on a subscale of the psychological resilience scale compared to the parents of children with other cancers (*p* < 0.05). The psychological scale scores of the parents of patients with advanced disease (high risk leukemia or metastatic solid tumor) were compared with the parents of patients with lower risk cancers. According to the risk status of cancer, there was no significant difference in the IUS, BHS, and PRS scores of the parents.

As a result of the analysis performed to compare the BHS and PRS scores of the parents according to the time elapsed after the cancer diagnosis, there was a significant difference only in the PRS scores (*p* < 0.01). It was observed that the resilience scores of the parents who had less than one month since the diagnosis of cancer were significantly higher than those who had passed 1–6 months and more than 1 year since the diagnosis. Future perception and social competence scores from the subscales of PRS differed significantly according to the time elapsed since diagnosis (*p* < 0.05). The future perception and social competence sub-dimension scores of parents whose children were diagnosed less than 1 month ago were statistically significantly higher than those who were 1–6 months and more than a year old. However, the total scores of BHS and IUS did not differ significantly according to the time elapsed since the child’s diagnosis (*p* > 0.05). Only the scores obtained from the “feelings about the future” subscale of BHS differ significantly according to the time elapsed after diagnosis (*p* < 0.05).

An interesting result of the analysis, significant differences were found only in the analyses performed for BHS, and the hopelessness scores of the parents whose child’s treatment was terminated were significantly higher than those whose active treatment continued (*p* < 0.05). Again, in the hope subscale of the BHS, it was observed that the parents whose child’s treatment was completed had significantly higher scores than the parents whose treatment was ongoing (*p* < 0.05). No significant difference was found between the parents of the children who received active treatment and the parents whose child’s treatment was completed in terms of IUS and PRS scores.

According to the demographic characteristics, age and income variables were found to have a positive relationship with PRS total score (*p* < 0.05). It means that psychological resilience of the parents who were older or had a higher income were found to be higher. It was determined that the variables of age, number of children, and income did not have a relationship with BHS (*p* > 0.05). There was a positive correlation between age and the scores of the IUS (*p* < 0.05), which means that older parents have a higher intolerance to uncertainty.

The mean scores of the hopelessness scale of the parents who had to quit their job during their child’s treatment was significantly higher than those who continued to work (*p* < 0.05). There was no significant difference between IUS and PRS scores of the parents who had to leave work during their child’s treatment compared to those who continued to work (*p* > 0.05). However, it was determined that the mean scores of the parents who left their jobs during the treatment in the “not knowing the future is disturbing” sub-dimension of the IUS were significantly higher than those who continued to work (*p* < 0.05).

## 4. Discussion

In our study, the most important findings are that the hopelessness scores are significantly higher among parents whose child’s treatment is terminated compared with those whose active therapy is ongoing; resilience scores are higher among parents who have less than 1 month since diagnosis of childhood cancer than later periods.

Having a child diagnosed with cancer, a potentially fatal disease, is one of the most stressful and life-changing experiences a parent can face [18]. The parent’s life becomes impacted by fear of death, treatment demands, side effects, financial burden, and a negative effect on family relations [19]. The diagnosis of cancer for their child contributes to the incidence of depression and anxiety disorders among parents [20]. Risk factors for distress in parents include disease severity, treatment intensity, negative affect, and insufficient personal resources and family stressors [11].

Some studies have reported that poor social support is associated with depression and hopelessness [21,22]. Hope helps in reducing the stress caused by cancer when used as a method of struggle. Hopelessness increases stress and negative expectations for the future. Resilience is the ability to quickly recover from disruptions in functionality resulting from stress assessments and return to previous functionality [23]. In our study, hopelessness, resilience, and intolerance to uncertainty were evaluated together among parents of children with cancer. It was observed that hopelessness was less in those with high psychological resilience.

In a recently published review on psychosocial interventions for families of children with cancer, 17 studies conducted to date were evaluated, and in these studies, distress, post-traumatic stress, and anxiety were evaluated on various scales [18]. In another review evaluating 48 studies performed between 1980 and 2005, similar tests were used to measure stress, depression, and anxiety in parents whose children were diagnosed with cancer [19]. In our study, different scales were used to assess stress and anxiety in parents of children with cancer, unlike previous studies. This study showed that hopelessness and intolerance to uncertainty were directly related to each other, and hopelessness and psychological resilience were negatively related. The study by Somasundaram and Devamani also confirms the results of the present study on the relationship between resilience and hopelessness [20]. They reported that resilience was significantly associated with less hopelessness and higher levels of perceived social support.

In a meta-analysis study, mothers and fathers of children newly diagnosed with cancer reported significantly greater stress levels than parents of healthy children, and mothers reported greater stress than fathers up to 12 months after diagnosis [9]. In another study, parents of children with cancer were evaluated in terms of post-traumatic stress symptoms (PTSS); no statistically significant difference was determined between mothers and fathers at the time of diagnosis [23]. A previous study reported that the younger age of children was significantly correlated with the severity of depression in the parents of children with cancer [21]. The present study observed no significant difference between mothers and fathers in the psychological scales. In our study, there was a positive correlation between the age of the child and the parents’ intolerance to uncertainty and psychological resilience. However, no significant correlation was determined with the age of the child in the hopelessness assessment of the parents.

In our study, while there was no significant change in the hopelessness and uncertainty intolerance scores of both mothers and fathers over time, psychological resilience scores were found to be higher in the first month and decreased over time. Manne et al. reported that depressive symptoms among parents of children with cancer and did not find any change between 1st, 3rd, and 6th months after the diagnosis [22]. Magal-Vardi et al. reported that in the 1st and 6th months of follow-up, the PTSS symptoms of the fathers decreased over time in proportion to the symptoms of the children, while there was no decrease in the PTSS of the mothers [23].

Magal-Vardi et al. also reported that no significant difference was determined between parents when PTSS symptoms were evaluated according to the risk group of children [23]. Similarly, in our study, there was no significant difference in terms of psychological resilience, hopelessness, and intolerance to uncertainty when parents were compared according to their children’s risk groups. However, no significant difference was found when parents compared their child’s diagnosis (leukemia vs. other cancers) in terms of psychological resilience, hopelessness, and intolerance to uncertainty. These results revealed that there was no difference in stress and anxiety levels between parents of children with cancer in terms of cancer type and risk group. It suggests that any cancer diagnosis in their children affects all parents similarly.

In this study, hopelessness was found to be higher in parents who left their jobs than those who continued to work after their child was diagnosed with cancer. Patiño-Fernández et al. suggested that families of children with cancer at risk for acute distress reactions needed psychosocial support at the time of diagnosis and throughout the treatment of cancer [24]. Sloper reported that psychological distress in parents remained high at 6 and 18 months after their child was diagnosed with cancer [25]. As an interesting result, our study showed that parents whose child’s treatment ended were more hopeless than parents whose child’s treatment was ongoing. This result suggested that parents could not return to their normal state psychologically after cancer treatment was over. Kazak et al. also emphasized that the importance of early identification of psychosocial risk factors to identify parents who were likely to need psychological support over time [14]. Additionally, Wikman et al. suggested that psychological distress was indicated to be a long-term problem for some parents who survived childhood cancer [26]. As a result of our study, it was observed that while psychological resilience was high in the first month of diagnosis, psychological resilience decreased at the end of the process. This result suggests that parents with high psychological resilience may need help later.

Psychological evaluation of parents of children with cancer at regular intervals at the time of diagnosis, during treatment, and at the end of treatment and support to those in need should be part of the multidisciplinary approach. We recommend routinely using psychological scales to determine need of psychological support for the parents as well as children.

### Study Limitations

The main limitation of this study is the cross-sectional design, which limits its ability to determine causality. This study surveyed a small sample of parents of children with cancer, but generalizability is limited by the fact that we have studied only parents of patients who are treated at our hospital. To the best of our knowledge, this is the first study to use different scales (BHS, PRS, IUS) to measure the level of stress and anxiety in parents of children with cancer [27,28,29,30,31,32,33,34,35,36,37]. Therefore, we were unable to compare the results of the scales with previous reports.

## 5. Conclusions

Regardless of the type and stage of cancer, there is no difference in the level of anxiety and stress among the parents of children with cancer. We suggest that parents who have children with cancer should be evaluated psychologically at regular intervals at the time of diagnosis, during treatment, and at the end of treatment, and that supporting those in need should be a part of the multidisciplinary approach.

## Figures and Tables

**Table 1:** Socio-demographic status of the parents of children with cancer.

**Gender**	***n***	**%**
Mother	57	67.86
Father	27	32.14
Age		
20–29	12	14.28
30–39	39	46.43
40–49	32	38.10
50–52	1	1.19
Marital status		
Married	79	94.05
Divorced	5	5.95
Job status		
Quit job	49	58.33
Continue job	35	41.66
Number of children		
1	16	19.04
2	34	40.48
3+	34	40.48
Income level		
TRY 0–2825	30	35.71
TRY 2826–3999	34	40.48
>TRY 4000	20	23.81

**Table 2:** Clinical properties of the patients.

**Age of the patient (years)**		***n***	**%**
0–6		42	50
7–12		24	28.57
13–19		18	21.43
**Type of Cancer**			
Leukemia	ALL	29	34.52
	AML	5	5.95
	Total	34	40.48
Solid Tumor	Neuroblastoma	10	11.90
	Rhabdomyosarcoma	10	11.90
	Osteosarcoma	6	7.14
	Hodgkin Lymphoma	6	7.14
	Germ Cell Tumor	3	3.57
	NHL	4	4.76
	Nasopharyngeal Carcinoma	2	2.38
	Wilms Tumor	3	3.57
	Ewing Sarcoma	1	1.19
	Brain tumor	3	3.57
	Optic Glioma	1	1.19
	CCRK	1	1.19
	Total	50	59.52
**Status of Metastasis**			
Metastatic		20	40
Non-metastatic		30	60
**Risk Status**			
Low risk		4	11.76
Intermediate risk		22	64.71
High risk		8	23.53

Abbreviations: ALL: Acute lymphoblastic leukemia, AML: Acute myeloblastic leukemia, CCCR: clear cell carcinoma of the kidney, NHL: Non-Hodgkin lymphoma.

**Table 3:** Correlation between Beck Hopelessness Scale and Psychological Resilience Scale and Intolerance of Uncertainty Scale.

		1	2	3
Beck Hopelessness Total Score (1)	r	1		
	*p*			
Psychological Resilience Total Score (2)	r	−0.560	1	
	*p*	0.000		
Intolerance of Uncertainty Total Score (3)	r	0.479	−0.412	1
	*p*	0.000	0.000	

Spearman Correlation.

## Data Availability

The data presented in this study are available upon reasonable request from the corresponding author. The data are not publicly available due to o ethical commitments for sensitive patient information.
